# Cleavage-stage human embryo arrest, is it embryo genetic composition or others?

**DOI:** 10.1186/s12958-022-00925-2

**Published:** 2022-03-17

**Authors:** Raoul Orvieto, Anat Jonish-Grossman, Sharon Avhar Maydan, Meirav Noach-Hirsh, Olga Dratviman-Storobinsky, Adva Aizer

**Affiliations:** 1 Infertility and IVF Unit, Department of Obstetrics and Gynecology, Chaim Sheba Medical Center (Tel Hashomer), 56261 Ramat Gan, Israel; 2 Sackler School of Medicine, Tel Aviv University, Tel Aviv, Israel; 3 The Tarnesby-Tarnowski Chair for Family Planning and Fertility Regulation, Sackler Faculty of Medicine, Tel-Aviv University, Tel Aviv, Israel; 4 Danek Gertner Institute of Human Genetics, Sheba Medical Center, 56261 Ramat-Gan, Israel

**Keywords:** Human embryos, Cleavage-stage, Blastocyst stage, CGH, Euploidy

## Abstract

Embryo transfer is a crucial step in IVF cycle, with increasing trend during the last decade of transferring a single embryo, preferably at the blastocyst stage. Despite increasing evidence supporting Day 5 blastocyst-stage transfer, the optimal day of embryo transfer remains controversial. The crucial questions are therefore, whether the mechanisms responsible to embryos arrest are embryo aneuploidy or others, and whether those embryos arrested in-vitro between the cleavage to the blastocyst stage would survive in-vivo if transferred on the cleavage-stage. We therefore aim to explore whether aneuploidy can directly contribute to embryo development to the blastocyst stage. Thirty Day-5 embryos, that their Day-3 blastomere biopsy revealed a single-gene defect, were donated by 10 couples undergoing preimplantation genetic testing treatment at our center. Affected high quality Day-3 embryos were cultured to Day-5, and were classified to those that developed to the blastocyst-stage and those that were arrested. Each embryo underwent whole genome amplification. Eighteen (60%) embryos were arrested, did not develop to the blastocyst stage and 12 (40%) have developed to the blastocyst stage. Nineteen embryos (63.3%) were found to be euploid. Of them, 12 (66.6%) were arrested embryos and 7 (58.3%) were those that developed to the blastocyst-stage. These figures were not statistically different (*p* = 0.644). Our observation demonstrated that the mechanism responsible to embryos arrest in vitro is not embryo aneuploidy, but rather other, such as culture conditions. If further studies will confirm that Day-5 blastocyst transfer might cause losses of embryos that would have been survived in vivo, cleavage-stage embryo transfer would be the preferred timing. This might reduce the cycle cancellations due to failure of embryo to develop to the blastocyst stage and will provide the best cumulative live birth-rate per started cycle.

## Introduction

Embryo transfer (ET) is a crucial step in IVF cycle, with increasing trend during the last decade of transferring a single embryo, preferably at the blastocyst stage. Despite increasing evidence supporting Day 5 blastocyst-stage transfer, the optimal day of embryo transfer remains controversial [1–5]. It has been suggested that one third to one half of human embryos produced by IVF do not develop to the blastocyst-stage in-vitro [6, 7].

In their Cochrane review on cleavage versus blastocyst stage embryo transfer in assisted reproductive technology (ART), Glujovsky et al. [3] have concluded that although there is an advantage favoring blastocyst transfer in fresh cycles, it remains uncertain whether the day of transfer impacts on cumulative live birth and pregnancy rates. Studies that favor Day 5 blastocyst-stage transfer usually report on outcomes per embryo transfer, rather than per cycle. While blastocyst transfer result in higher implantation rate, cleavage-stage transfer is associated with higher numbers of embryos available for freezing, and lower cancellation rate due to no embryos available for transfer [8]. Therefore, since developmental arrest during culture to the blastocyst stage might impair live birth rate, comparison should be analyzed with reference point cycle start, and not, with embryo transfer.

Culturing cleavage-stage embryo on Day 3 to the blastocyst-stage will select the developing, while discarding the non-viable embryos. Embryos usually arrest for various reasons, such as culture conditions, poor metabolism or DNA damage [9]. Whether aneuploidy can directly contribute to embryo development to the blastocyst stage remains unclear.

For better understanding of the mechanisms of embryo development, and whether aneuploidy can directly contribute to embryo development to the blastocyst stage, Qi et al. [10] examined the chromosome integrity of blastocysts and arrested embryos that did not develop to the blastocyst stage, from patients undergoing IVF and preimplantation genetic testing for aneuploidy (PGT-A). Most of arrested embryos were aneuploid (44/45), compared to 32/45 of the developing embryos (blastocysts) (32/45, *P* < 0.01). Both euploid (92.9%) and aneuploid (42.1%) embryos developed to blastocyst- stage.

The crucial questions are therefore, whether the mechanisms responsible to embryos arrest are culture conditions or embryo aneuploidy, and whether those embryos arrested in-vitro between the cleavage to the blastocyst stage would survive in-vivo if transferred on the cleavage-stage? Aiming to shed more light on these questions, we assessed the ploidy of top quality cleavage-stage embryos that were cultured to day-5, either arrested at the cleavage-stage or developed to the blastocyst-stage.

### Patients and methods

Day-5 embryos, in which their Day-3 blastomere biopsy revealed a single-gene defect, were donated by couples undergoing PGT treatment at our center, from May 2021 to September 2021. All embryos were of high quality before the Day-3 biopsy, e.g. 7–8 equal blastomeres with no fragmentations. Affected embryos were cultured to Day-5 in the same condition, and were classified to those that developed to the blastocyst-stage and those that were arrested. Each embryo underwent whole genome amplification (WGA).

#### "WGA

Full-genomic amplification of the DNA was carried out by WGA-PCR PicoPlex SingleCell WGA Kit (Rubicon Genomics) [Takarabio. GenetiSure Pre-Screen Complete Protocol. Available from: https://www.takarabio.com/assets/documents/User%20Manual/ PicoPLEX%20WGA%20 Kit%20Protocol-At-A-Glance-070717.pdf.]. The quality and quantity of DNA received during amplification were controlled by electrophoresis using 1% agarose gel.

#### Array-CGH

WGA products were processed referring to the protocol of Agilent oligonucleotide array-based CGH for single cell G4410–90,003 Revision B0, October 2018. These products were fluorescently labelled with controls (Human Reference DNA Female/Male) according to the instructions of SureTag Complete DNA Labeling Kit (Agilent technologies, CA, USA), and then competitively hybridized to G9500A GenetiSure Pre-Screen Complete kit (8 × 60) Agilent technologies, CA, USA) [Agilent. Oligo aCGH/ChIP-Chip Hybridization Kit Protocol. Available from: https://www.agilent.com/en/product/cgh-cgh-snp-microarray-platform/cghcgh-snp-microarray-kits-reagents/oligo-acgh-chip-on-chip-hybridizationkit-228433]".

#### Interpretation of array-CGH results

Data analysis was accomplished according to the manufacturer recommended single cell analysis method. We only reported whole chromosome monosomies or trisomies. The operator of the molecular analysis (AJG) was blinded to the samples’ sources.

Informed consent was obtained from all patients before participation in the study, and the study was approved by our Institutional Clinical Research Committee (IRB SMC-19–6140). The study required no modification of patient’s routine follow-up or treatment.

## Results

Ten women (age 31.7 ± 3.4 yrs, range 27–39 yrs) have donated 30 high-quality Day-3 embryos, which were found to be affected following Day-3 blastomere biopsy, and were cultured to the balastocyst-stage. Of which, 18 (60%) were arrested, did not develop to the blastocyst stage and 12 (40%) have developed to the blastocyst stage. Nineteen embryos (63.3%) were found to be euploid. Of them, 12 (66.6%) were arrested embryos and 7 (58.3%) were those that developed to the blastocyst-stage. These figures were not statistically different (*p* = 0.644). The CGH results of the embryos are presented in Table 1.

**Table 1:** The CGH results of the study embryos

Patient	Age (yrs	Embryo #	Arrested in Day 5	Blastocyst in day 5	Day 5 ploidy
A	32	1	X		Euploid
B	28	2	X		Euploid
C	35	3		X	Euploid
C		4		X	Aneuploidy
C		5		X	Aneuploidy
C		6	X		Euploid
C		7	X		Euploid
C		8	X		Euploid
D	34	9	X		Aneuploidy
D		10	X		Aneuploidy
E	30	11	X		Euploid
F	32	12	X		Aneuploidy
F		13	X		Euploid
G	39	14		X	Aneuploidy
G		15	X		Aneuploidy
H	27	16		X	Aneuploidy
H		17		X	Euploid
H		18		X	Aneuploidy
H		19	X		Aneuploidy
H		20		X	Euploid
I	29	21		X	Euploid
I		22	X		Euploid
I		23		X	Euploid
I		24	X		Euploid
I		25	X		Aneuploidy
I		26		X	Euploid
I		27	X		Euploid
J	31	28	X		Euploid
J		29		X	Euploid
J		30	X		Euploid

## Discussion

In the present study of extended embryo culture, only 40% of good quality Day-3 embryos developed to the blastocyst stage. Figure that is in accordance with the reported figures in young patient [11]. Moreover, 11 embryos (63.3%) were found to be euploid. Again, figure that is in agreement with both the STAR study [12] of patients' age 33.7 ± 3.59yrs, and the study by Yan et al. [13] of patients' age 29.1 ± 3.6 yrs, with euploidy rate of good-quality blastocysts of 43.1%, and 69.8%, respectively.

One of the interesting observation obtained in the present study is the comparable (*p* = 0.644) euploidy rates between arrested embryos (66%) and those that developed to the blastocyst-stage (58%). These figures are reassuring and differ from those demonstrated by Qi et al. [10]. In the later, only 15.5% of all embryos were euploid, and 97.8% and 71.1% of the arrested embryos and the developing embryos (blastocysts) were aneuploid, respectively. These higher rates were explained and related to small sample size, and the delay between stopping to develop (Day 3) and the biopsy of the arrested embryos (Day 6), which might be the culprit of the DNA degeneration or damages. In the present study, euploidy rate in all the embryos and in the different subgroups (arrested and developing embryos) did not differ from those reported in the literature [12, 13] in young patients, ranging between 43.1% and 69.8%. We might therefore speculate that the cause of developmental arrest is not the embryo genetic integrity, but rather other reasons, such as the culture conditions. Moreover, since in the present study most embryos failing to progress to the blastocyst stage were chromosomally normal, we believe that in different conditions, they would progress to the blastocyst- stage and result in a live birth if transferred on Day-3.

Reinforcement to the aforementioned speculation might be found in the study by Xiao et al. [14], who uniquely examined the pregnancy rate per embryo in patients with only one surviving embryo. Day 3 embryo transfer resulted in significantly higher clinical pregnancy and live birth rates, when both adjusted and unadjusted for embryo grading. Notwithstanding that this advantage was observed despite the lower-quality of embryos transferred at the cleavage-stage, as compared to those cultured and planned to be transferred at the blastocyst-stage. It might be therefore concluded, that cleavage-stage embryos that arrest during in-vitro culturing to the blastocyst-stage, might still have the ability to implant if transferred on Day-3. It might be therefore possible that the developmental arrest of cleavage-stage embryos in vivo is lower than their attrition in vitro, and that blastocyst transfer leads to the loss of embryos that may have survived in vivo. It should be emphasized, that culturing cleavage-embryos to the blastocyst-stage is a selection measure, not treatment.

While the reported prevalence of mosaicism in human cleavage- and blastocyst-stage embryos, based on PGT-A, ranges from 50% to up to 90% [15], there is substantial evidence that an embryo’s chromosome complement might change throughout preimplantation development [16], with lower levels of mosaicism in blastocyst-stage, as compared to Day 3 embryos [17]. Moreover, "mosaic" embryos demonstrate increased cell proliferation and cell death in comparison to euploid embryos, observations suggestive of significant self-correction abilities of embryos [18, 19]. At the same time, Shahbazi et al. [20] have recently characterized the development of embryos with different specific aneuploidies up to day 9 and uncovered tissue-specific alterations. While some aneuploidies developed similarly to euploid embryos, others exhibited high rates of developmental arrest, endorsing the genetic plasticity that exists at preimplantation stages in human embryos and the possibility that, while chromosomal abnormalities do not appear to play a significant role in embryo arrest, some cases of embryos arrest due to chromosomal abnormalities cannot be ruled out.

The limitations of our study is the small sample size of affected embryos donated for the study, which are strongly influenced by the ethical concerns.

## Conclusion

We might be therefore concluded, that in good prognosis patients (young patients with optimal ovarian response and high number and quality of embryos [11], transferring a fresh blastocyst might shorten the time to pregnancy, compared with cleavage-stage embryo transfer, probably without increasing the cumulative live-birth rate per started cycle [3]. Moreover, in unselected patients, or those with sub-optimal or poor ovarian response, cleavage-stage embryo transfer will reduce the incidence of cycle cancellation due to failure of embryo development to the blastocyst stage and will provide the best cumulative live birth-rate per started cycle. At our center, transferring embryos at the blastocyst-stage is offered to patients with > 8 Day-3 cleavage stage embryos. Further large well-designed studies are required to validate our observation.

## Data Availability

Not applicable.
